# HLA-DR Genotyping and Mitochondrial DNA Analysis Reveal the Presence of Family Burials in a Fourth Century Romano-British Christian Cemetery

**DOI:** 10.3389/fgene.2017.00182

**Published:** 2017-12-05

**Authors:** Canh P. Voong, Patrick S. Spencer, Cristina V. Navarrete, David Turner, Soren B. Hayrabedyan, Philip Crummy, Emma Holloway, Mike T. Wilson, Patricia R. Smith, Nelson Fernández

**Affiliations:** ^1^School of Biological Sciences, University of Essex, Colchester, United Kingdom; ^2^Histocompatibility and Immunogenetics Laboratory, NHS Blood and Transplant, London, United Kingdom; ^3^Division of Infection and Immunity, Faculty of Medical Sciences, University College London, London, United Kingdom; ^4^Histocompatibility and Immunogenetics Laboratory, Scottish National Blood Transfusion Service, Royal Infirmary of Edinburgh, Edinburgh, United Kingdom; ^5^Institute of Biology and Immunology of Reproduction, “Akad. Kiril Bratanov,” Bulgarian Academy of Sciences (BAS), Sofia, Bulgaria; ^6^Colchester Archaeological Trust, Colchester, United Kingdom; ^7^MAPI Group, Ontario, Canada

**Keywords:** extramural fourth century Romano-British cemetery, family groupings, HLA-DR, mtDNA, Colchester (Camulodunum)

## Abstract

In Colchester, Britain's oldest recorded town, during the Roman period there were areas which were clearly used solely as cemeteries. One of the most significant is at Butt Road, which includes a late Roman probable Christian cemetery with an associated building, apparently a church, that overlies and developed from a pagan inhumation cemetery. DNA was extracted from the long bones (femurs) of 29 individuals, mostly from a large complex of burials centered on two timber vaults. These were thought to comprise a number of family groupings, deduced from osteological analysis, stratigraphical and other considerations. The use of a modified version of the silica-based purification method recovered nanogram quantities of DNA/gram of bone. Two-stage amplification, incorporating primer-extension preamplification-polymerase chain reaction, permitted simultaneous amplification of both mitochondrial and nuclear DNA. Sequence-specific oligonucleotide probes yielded human leukocyte antigen (HLA)-DR typing of seven samples, with four revealing the infrequent HLA-DR10 genotype. Examination of the control region of mitochondrial DNA (mtDNA) by direct sequencing revealed polymorphisms yet to be reported in the modern population. HLA-DRB typing and mtDNA analysis affirmatively supported kinship among some, if not all, individuals in the “vault complex” and demonstrate a continental European origin of the individuals investigated.

## Introduction

We conducted an ancient DNA (aDNA) study of skeletons from the fourth century extramural Romano-British inhumation cemetery at Butt Road in Colchester, Essex, U.K., interpreted as probably an early Christian cemetery (Crummy et al., 1993). Colchester (Camulodunum) served as the first *colonia* of the Roman province of Britannia, known as Colonia Victricensis. The settlement's residents initially comprised retired veteran soldiers.

**Figure 1:**
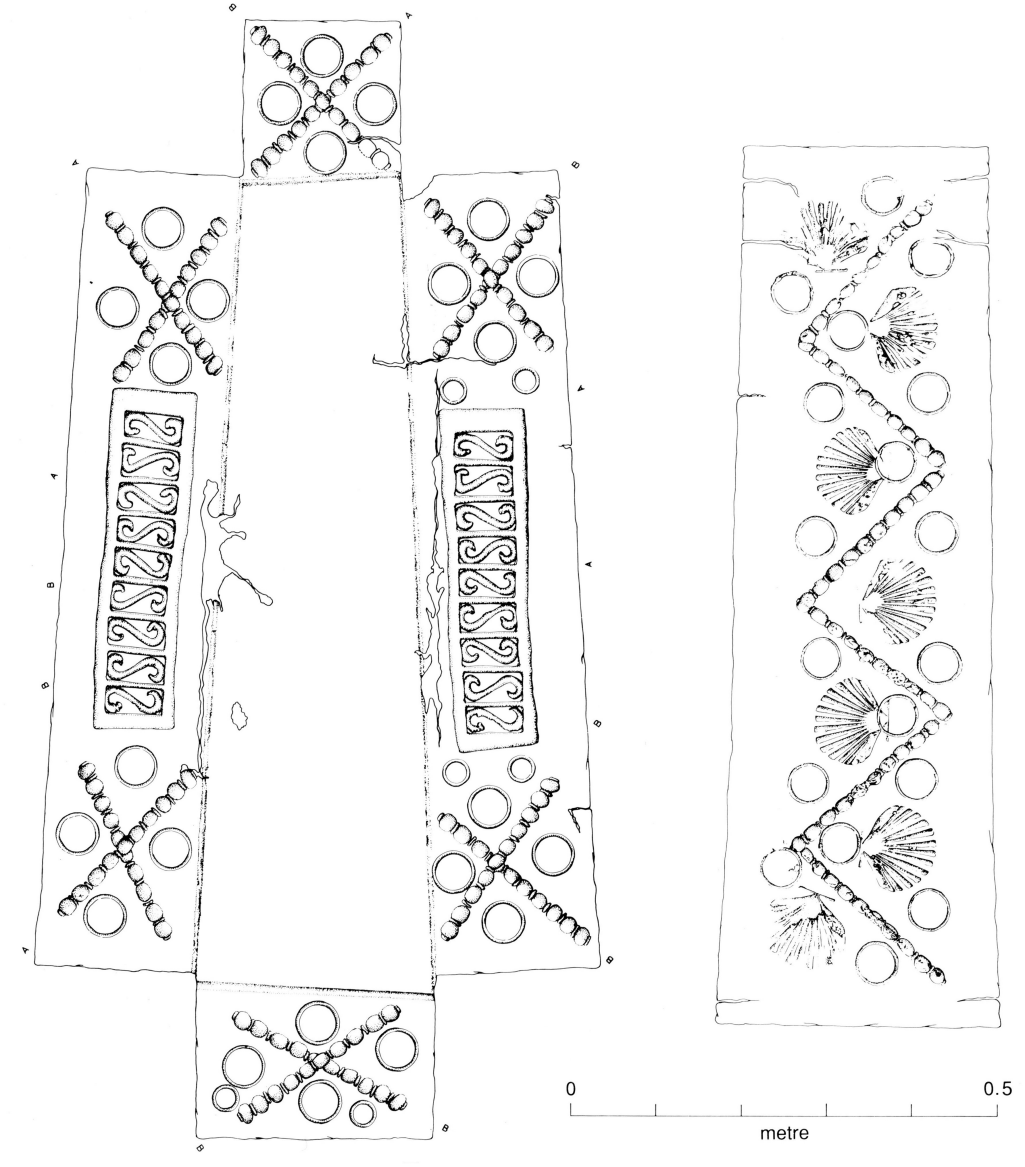
Decorated lead inner coffin, Period 2 (Figure 2.69, Crummy et al., 1993). One of two decorated lead inner coffins from Butt Road Period 2. While the Butt Road decorated lead coffins are not indisputably Christian, their decoration is paralleled in a Christian context.

The Butt Road cemetery, one of the largest excavated Romano-British inhumation cemeteries, consists of two separate but superimposed cemeteries designated Period 1 and 2, Period 1 being further subdivided into three phases. Period 1 consisted of 61 graves primarily from the mid-third century AD. In the final phase of Period 1 (Phase 3; 300-320/40 AD) most of the site was set aside for a formally established pagan inhumation cemetery, the coffined graves aligned north-south, with some clearly defined boundaries and family plots. The Period 2 cemetery contained at least 620 burials of fourth century date; it is distinguished from Period I by stratigraphical differences and changes in mortuary treatment (Crummy et al., 1993). Six timber vaults (or boxes) for double and single burials, dated around the middle of the fourth century (Hatton, 1999), were also found in the Period 2 cemetery. Most notably in Period 2, the internments are oriented east-west. The change in alignment was fairly sudden and is considered likely to mark the adoption of a Christian burial rite, apparently concomitant with the construction of an apsidal building, probably a church, possibly serving also as a martyrium (Crummy et al., 1993), at the north-west edge of the cemetery. The Christian identity of the Period 2 level may be seen as strengthened by the incidence of neonatal burials, strict cemetery organization, the absence of grave goods, except those denoting the status of the individual (particularly vessels and coins), and protection of the body with a lining or packing (Crummy et al., 1993). Period 2 at Butt Road contained three lead-lined coffins and a number of plaster burials, which, along with the timber vaults, represent specialized minority rites. Two lead inner coffins were decorated (Figure 1), bearing symbols (circle, zig-zag, monogrammatic cross) paralleled in an undoubted Christian context (Crummy et al., 1993). Based on coin evidence, the first phase of the apsidal construction at Butt Road is dated as ca. 320-340 AD, which is taken to be the starting date for Period 2. The Period 2 cemetery thus existed not more than a decade or so after the Edict of Milan in 313 AD, an agreement recognizing the rights of Christians within the Roman Empire, enabling them to practice their faith in recognizable churches.

During the major phase of the excavation at Butt Road (between 1976 and 1988), most unusually the excavators were able to postulate the existence of family groups in the Period 2 level. These were inferred from criteria including spatial relationships between some of the burials and non-metrical traits of the skull and post-cranial skeleton. Most of the putative family groups were located close to two closely opposed vaults, CF55 (vault I) and grave (G) 366 (vault II), in the south central area of the cemetery. The other groups were often small, occasionally comprised just of pairs of burials, two of which were close to another vault (G225). CF55 and G366 were surrounded by a densely-packed clustering of over 30 later burials, referred to as the “vault complex” (Crummy et al., 1993). It included a significant number of graves of neonatal or very young infants. The density of graves around and between the two vaults suggests that those interned in the vaults were individuals of special importance.

The late Romano-British cemeteries tend to display a greater degree of internal organization and standardization in burial than earlier cemeteries (Hatton, 1999). It is thought that both increased bureaucratic control and the dissemination of Christian belief played a part in this trend (Sparey-Green, 2006). This notwithstanding, there is evidence to indicate that control by family custom over burial became more prominent during the fourth century. For example, in addition to the postulated family burials at Butt Road, there is evidence for one or more family plot at Poundbury (Dorchester), Verulam Hills Field (Verulamium), Lankhills (Winchester) and, possibly, at London (Eastern Cemetery), Gloucester (Kingsholm Road and Parry Lodge Site) and Kelvedon (Area J) (Hatton, 1999). However, a major problem has been the authentication of the supposed family burials, since, to date, the criteria used to identify them have not been, on their own, definitive. This study aimed to determine kinship relationships, by DNA sequence analysis, between Butt Road Period 2 skeletons believed to be biologically related. In addition, as part of this study, we sought to identify genetic correlations between ancient and modern populations. HLA-DRB1 genotyping and mitochondrial DNA (mtDNA) analyses were performed. The approach of combining HLA typing of human aDNA with bioarchaeology is seldom applied.

## Materials and methods

### Permission to excavate and removal of buried human remains

The Essex County Council, the landowner of the Butt Road site, sanctioned the Butt Road excavation and provided financial and other support for the project. The Butt Road human remains were assessed as contributing toward further scientific understanding. Accordingly, a coroner's license was obtained to allow their removal and storage at Colchester and Ipswich Museums.

### Skeletal material

Long bones (femurs) from Butt Road Period 2 graves, representing 26 human individuals, were studied. In preparation for molecular genetic analysis, these were removed from graves *in situ*, under DNA-free conditions. The individuals sampled were all those hypothesized to constitute family groupings, based on criteria that included stratigraphical association, the idiosyncratic distribution of some types of grave good and non-metric and congenital variants on the skeletons (see Supplementary Information). The state of skeletal preservation ranged from poor to good. Twelve skeletons comprised four presumed family graves (Groups C, O, P), within the complex around the vaults CF55 and G366 (Figures SI1, SI2 in S1 Text). Two other skeletons from this complex, also sampled (G346, G377), formed part of a sequence of four graves. Eleven skeletons were representative of four other hypothesized family groupings. Two (G204 and 224) were in graves adjacent to another vault (G225).

The age and sex of each skeleton had been inferred from osteological measurements (Crummy et al., 1993). For gender determination, priority was given to innominate morphology. The samples used and morphological data are shown in Table SI4.

### Extraction of DNA from bone samples

The samples were retrieved DNA-free. Pre-extraction, post-extraction, and electrophoresis work were carried out in separate laboratories. Potential contaminating DNA was removed from the bone samples by removing the top 1–2 mm of the bone surface with a sandblaster. The bones were then exposed to UV light.

The extraction protocol used was a modified version of that developed and described by Boom et al. (1990) and subsequently adapted by Höss and Pääbo (1993) for extracting DNA from bones. The technique utilizes silica beads and the chaotropic agent guanidinium thiocyanate (GuSCN). During initial extractions, decalcification of the bones was employed. Powdered bone was added to 50 volumes of 0.5M EDTA, pH 8.5, and incubated at room temperature on a tube rotator for 72 h with two changes of EDTA. During later extractions, the decalcification step was omitted, as DNA was being lost. The bone powder was incubated with an extraction buffer (5M GuSCN, 1.3% Triton X-100, 0.1M Tris-HCl pH6.4, 0.02M EDTA pH8.2) for 12 h at 55°C and 250 RPM in an orbital shaker. The stoichiometry of bone powder to extraction buffer was 1 g−2 ml.

Following incubation, the mixture was centrifuged for 5 min at 12,000 g. The supernatant was removed in 500 μl aliquots and added to a mixture of 250 μl of extraction buffer and 20 μl silica suspension prepared as described by Boom et al. (1990). This mixture was incubated in a tube rotator for 1 h at room temperature. It was then centrifuged at 13,000 g for 2 min, after which the silica pellet was washed and resuspended by vortexing in 250 μl of washing agent (250 mL, ethanol, acetone). The suspension was centrifuged for 1 min at 13,000 g, the supernatant was discarded and the washing step repeated; twice with 96% ethanol and once with acetone.

After the final wash, the silica pellet was dried in a heat block (Techne Dri-Block) at 56°C for 10 min. Maintaining the temperature at 56°C, the DNA was eluted by adding 35–150 μl of Purite water. The pellet was briefly vortexed, incubated for a further 10 min and then centrifuged for 5 min at 13,000 g. The resulting supernatant containing the DNA was stored at −20°C. The elution process was carried out either once or twice. Negative control extractions containing everything except the bone powder were performed in parallel to detect contamination in the reagents.

### Quantitation of aDNA

A fluorescence assay using the PicoGreen dsDNA quantitation reagent (Molecular Probes) was used to determine the concentration of DNA in the extracts. Working solutions of PicoGreen were prepared by diluting the stock solution 1:200 with TE (10 mM Tris-HCl, pH 7.5; 1 mM EDTA) and protected from light to prevent photobleaching. Aliquots of DNA extract (60 μl) were diluted with TE to a final volume of 1 ml with the PicoGreen working solution. The samples were excited at 480 nm and the fluorescence intensity was read from 500 to 600 nm using a fluorometer. The efficacy of the silica-based extraction technique was investigated by adding known amounts of DNA (0.2–1.0 μg of 1 kb ladder) to the extractions (see Supplementary Information).

### PCR amplification

The extracted DNA was amplified using primer-extension preamplification (PEP)-PCR (PEP-PCR). The method of Zhang et al. (1992) was used. Four sets of mitochondrial primers (Table SI6) were used to amplify the first hypervariable segment of the mitochondrial control region. To amplify it, an aliquot (10 μl) of PEP-PCR product was added to the PCR mixture, consisting of mitochondrial primers (0.4 μM), dNTPs (200 μM of each), mixed dNTPs (200 μM), PCR buffer (5 μl of 10X stock with 1.5 mM MgCl_2_), Q-solution (Qiagen, 10 μl of 5X stock) and Taq DNA polymerase (1.0 μl, 5U), made up to 50 μl with Purite water. An aliquot (35 μl) of mineral oil, added on top of the PCR mixture, prevented evaporation. DNA from Raji cells was used as positive control. Negative controls containing no DNA, and the extraction negative control, were performed in parallel. DNA from C.P. Voong, who alone handled the bone samples, was also typed; his HLA phenotype differed from DRB1 (data not shown). Potential contamination with DR3/DR10 (Raji cell line) was ruled out.

The PEP-PCR samples were placed in a thermal cycler (Techne Cyclogene) for one cycle at 94°C, for 5 min, followed by 50 cycles of denaturation (1 min at 94°C), annealing (2 min at 37°C), extension (4 min at 55°C).

PCR amplification of HLA-DR DNA was performed as above for mtDNA. HLA-DR primers (0.4 μM) that flanked the first, first and second, and the third hypervariable regions were used (Table SI6). All mtDNA, HLA-DRB, and amelogenin results were replicated from at least two independent extracts and PCR amplifications.

To improve the chances of successfully amplifying single copy nuclear genes, improvements were made to the extraction procedure. Prior to extraction, collagenase was used to remove collagen (see Supplementary Information). The extracts were then pooled and concentrated using micro-concentrators with a 30kd exclusion membrane. The concentrated extracts were added to PEP-PCR and wax mediated hot start was implemented; 6.25 U of Taq was required during PEP for subsequent specific PCR to succeed. An aliquot (10 μl) of the PEP-PCR products was added to the specific PCR mixture, containing primers encompassing the first and second hypervariable region.

### Gender determination

Genetic sex was determined for 14 samples by amplifying a segment of the XY homologous amelogenin gene. A pair of primers was synthesized that flank part of the amelogenin first intron, covering 106 and 112 bps of the X and Y homologs, respectively (Gaensslen et al., 1992). For the amplification, an aliquot (5 μl) of PEP-PCR product was added to the PCR mixture, comprised of the gender determining (amelogenin) primers (0.4 μM), PCR buffer (2.5 μl of 10X stock with 15 mM MgCl_2_), Q-solution (5 μl of 5X stock) and Taq DNA polymerase (0.5 μl, 5U). With Purite water added, the total PCR volume used was 25 μl. BSA was added and wax mediated hot start employed. Negative controls for PCR and extractions were performed in parallel.

### Gel electrophoresis

Amplified DNA samples were electrophoresed using low melting point agarose at 100 V for 45 min. For smaller fragments of DNA, or when two fragments of similar size needed to be separated, polyacrylamide gel electrophoresis was used. Samples were electrophoresed at 200 V for 45 min, or until the bromophenol blue dye front had reached the bottom. The DNA was detected on the agarose or polyacrylamide gel by staining the gels with ethidium bromide or SYBR gold.

The methodology used to recover DNA fragments from agarose gels was based on that described by Sambrook and Russell (2001).

The samples were cleaned using centricon 30 spin columns followed by sequencing in both directions with the appropriate primers. A Taq DyeDeoxy terminator sequencing kit (Perkin Elmer Applied Biosystems) was used, following the manufacturer's instructions. The sequence reactions were analyzed on an automatic sequencer (ABI 377).

### PCR HLA-DRB molecular typing

To determine HLA-DR types, sequence-specific oligonucleotide probes (SSOP) were used to detect polymorphic sequences in the second exon of the HLA-DRB gene. Two methods were used to HLA type the amplified DNA. The first used the kit INNO-LiPA (Immunogenics), which uses the reverse hybridization principle; that is, the probes were immobilized on nylon membranes and the DNA added to the probes (Saiki et al., 1989). The second technique used was PCR-SSOP (see Supplementary Information), wherein the PCR product is fixed on the membrane and probes are added later.

## Results

### Amplification of mitochondrial and nuclear DNA

Table SI8 lists the PCR results obtained from the samples extracted. Amplification products encompassing the first hypervariable segment within the control region of the mitochondrial genome were obtained for seven samples. Ten samples were positive for HLA-DRB and eight for gender-determining PCR.

### INNO-LiPA HLA DRB typing

HLA-DRB1 typing using the INNO-LiPA kit was performed on PEP samples from G390, G396, G370, G358, G346, and G298. Some samples typed had already given positive PCR products with mitochondrial primers. However, despite this, and the positive PEP-PCR, the results were all negative (data not shown). The conditions for typing were optimal, however, since the built-in positive control yielded a correct result.

### HLA-DRB typing using polymerase chain reaction and sequence specific oligonucleotide probes (PCR-SSOP)

#### Amplifying HLA-DRB1

The suspected reason for the failure of HLA-DRB typing using INNO-LiPA is the distance between the primers; the PCR primers used in the INNO-LiPA assay give products of 300-bp and greater. Therefore, for the PCR-SSOP analysis, primers were designed that amplified a smaller region (123-bp) than was required for INNO-LiPA typing (Table SI6). These flanked both the first and second hypervariable region of the second exon of the HLA-DRB gene, polymorphic sites confined to a short region of the gene.

**Figure 2:**
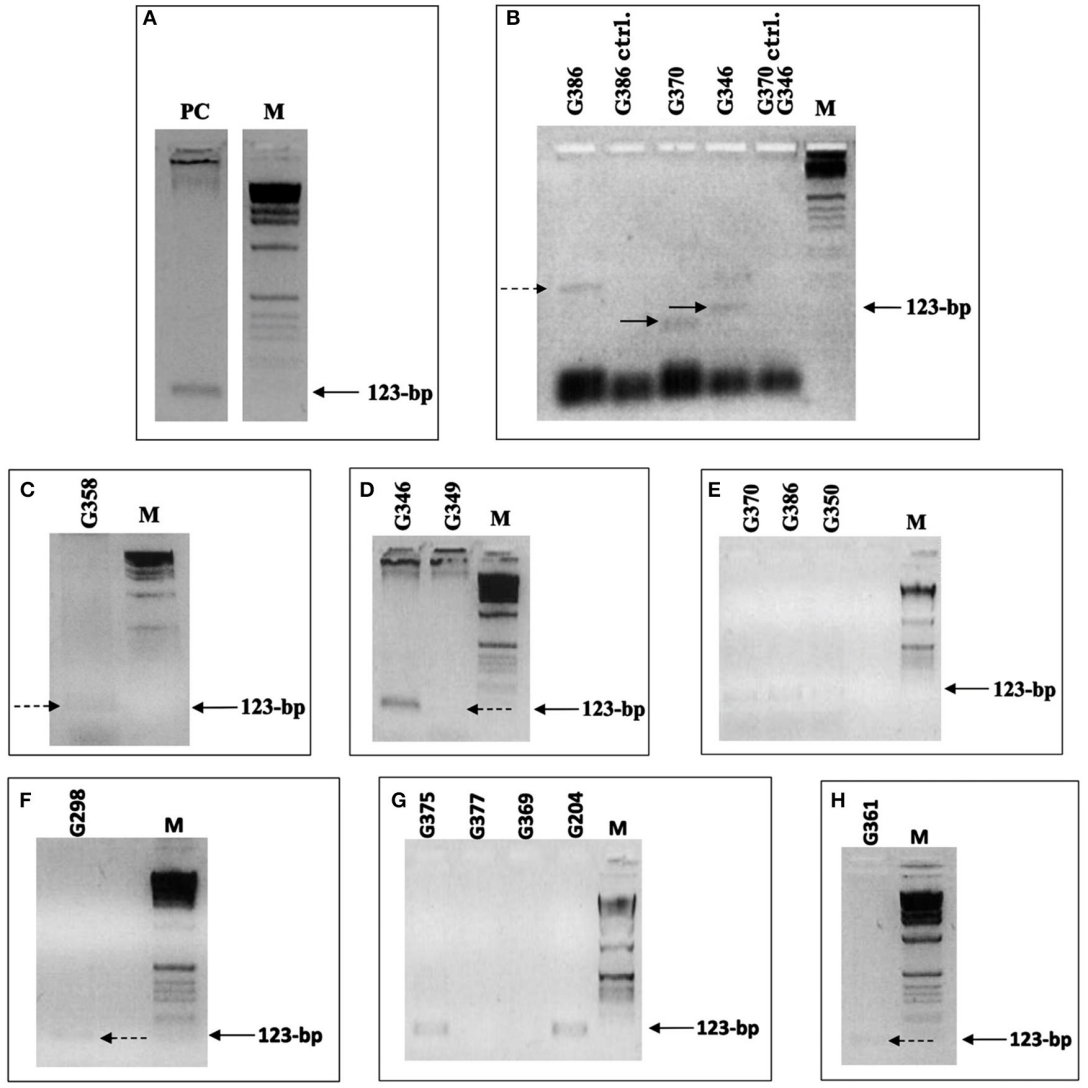
HLA-DRB PCR. **(A–H)** Samples that yielded HLA-DRB products. Primers 1 and 3 were used, flanking the first and second hypervariable region, giving a 123-bp product. **(A)** DNA (extracted from C.P. Voong) used for the positive control to indicate that the primers and PCR conditions were adequate to give specific amplifications. **(B)** Positive primer-extension pre-amplification (PEP) PCR test samples and negative extraction control samples (ctrl.), which were extracted simultaneously. The samples were added to PCR with the annealing temperature set at 53°C. Only one of the samples gave a band of the expected size. **(C–H)** Positive HLA-DRB PCR results, achieved after modification of the extraction protocol and after concentration of DNA extracts. **(C)** Sample G359 positive result, achieved after extracted DNA was concentrated from 500 to 50 μl. **(D)** Samples G349 and G350, which were extracted together using 2 × 3 and 2 × 4 g of bone powder, respectively, and were pooled together. Carrier DNA was used during extraction on samples G386 **(E)**, G298 **(F)**, and G361 **(H)**. Dashed and solid arrows indicate the position of PCR products that are extremely weak but present on the gel. M is the 1 kb molecular weight marker used.

To test HLA-DRB primers 1 and 3 (Table SI6) and the PCR cycling conditions, DNA extracted from blood was used to determine whether specific amplification was achieved. A clearly defined band of the expected size was visible on the agarose gel, indicating that the primers and PCR conditions were adequate to give specific amplifications (Figure 2A).

Next, an aliquot (10 μl) of positive PEP-PCR product from the ancient samples was added to a PCR mixture with the DRB primers flanking both first and second hypervariable region. Initially, samples G386, G370, and G346 were investigated, applying 35 cycles, with the annealing temperature set at 53°C. Single bands of varying length were visible on the gels [G370 (lane 4) shows a band <123 bp and G386 (lane 1) shows a band >123 bp]; only one of the samples gave a band of the expected size (Figure 2B). Thus, under these PCR conditions, different amplicons were obtained from the same sample. The negative extraction controls were correct, however, suggesting the bands were not produced from primers annealing to one another and extending.

With the modifications made to the extraction protocol and the concentrating of the extracts, 10 samples yielded positive HLA-DRB PCR results (Figures 2C–H). The success of amplification varied from sample to sample. A positive result was observed for sample G358 (Figure 2C) only after several attempts. The extract was concentrated down from 500 to 50 μl with some difficulty. PEP-PCR of the sample G358 was negative even when 7.5U of Taq was used and subsequent specific HLA-DRB PCR was also negative. It was suspected that during concentration of the extract, the inhibitors were also being concentrated. To overcome this inhibition, 10U of Taq and wax mediated hot start was used. The result was an extremely faint band suggesting amplification had taken place despite being severely hindered. Samples G349 and G350 were extracted together using 2 × 3 and 2 × 4 g of bone powder, respectively. The extracts were pooled together and concentrated, as already described. Positive PEP products were faintly detectable and subsequent PCR resulted in positive amplification.

The PCR products when electrophoresed through an agarose gel showed a bright band for sample G346 and a very weak band for G349 (Figure 2D). The brightness of the band for G346 suggests there may have been contamination. However, as the negative control was negative, the sample was kept and typed.

#### SSO probes

Twelve SSO probes were designed/selected to give the major serological types (Table SI7). The probes were biotinylated during synthesis. Eight of the probes used were complementary to the polymorphic sites within the first hypervariable region. To eliminate the possibility of inadequate typing conditions or that not enough templates dotted onto the membrane, the probe DR ALL, which is positive for all DR alleles, and a further probe DR 5704 (DR 14 specific), were also used. Probe DR 1003 was positive for many of the samples typed. This probe is positive for three serologic determinants, DR 3, 6, and 11. To separate DR 3, 6, and 11 it was necessary to target another region of the DRB gene where the types can be distinguished. It was decided the third hypervariable region would be the best region to amplify and primers were designed accordingly (Table SI6). Four of the probes, DR 7004, DR 5703, DR 5704, and DR ALL, hybridized to the third hypervariable region (Table SI7).

To distinguish between DR3, 6, and 11, three probes were used together with a positive control which hybridizes to all DR types. The three probes were specific for DR3, DR11, and DR14. DR6 is subdivided into DR13 and DR14. A probe for DR6 could not be designed around the regions amplified; therefore a negative result will be interpreted as DR6. In an ideal situation, a positive indication would be preferred to ascertain a type.

**Figure 3:**
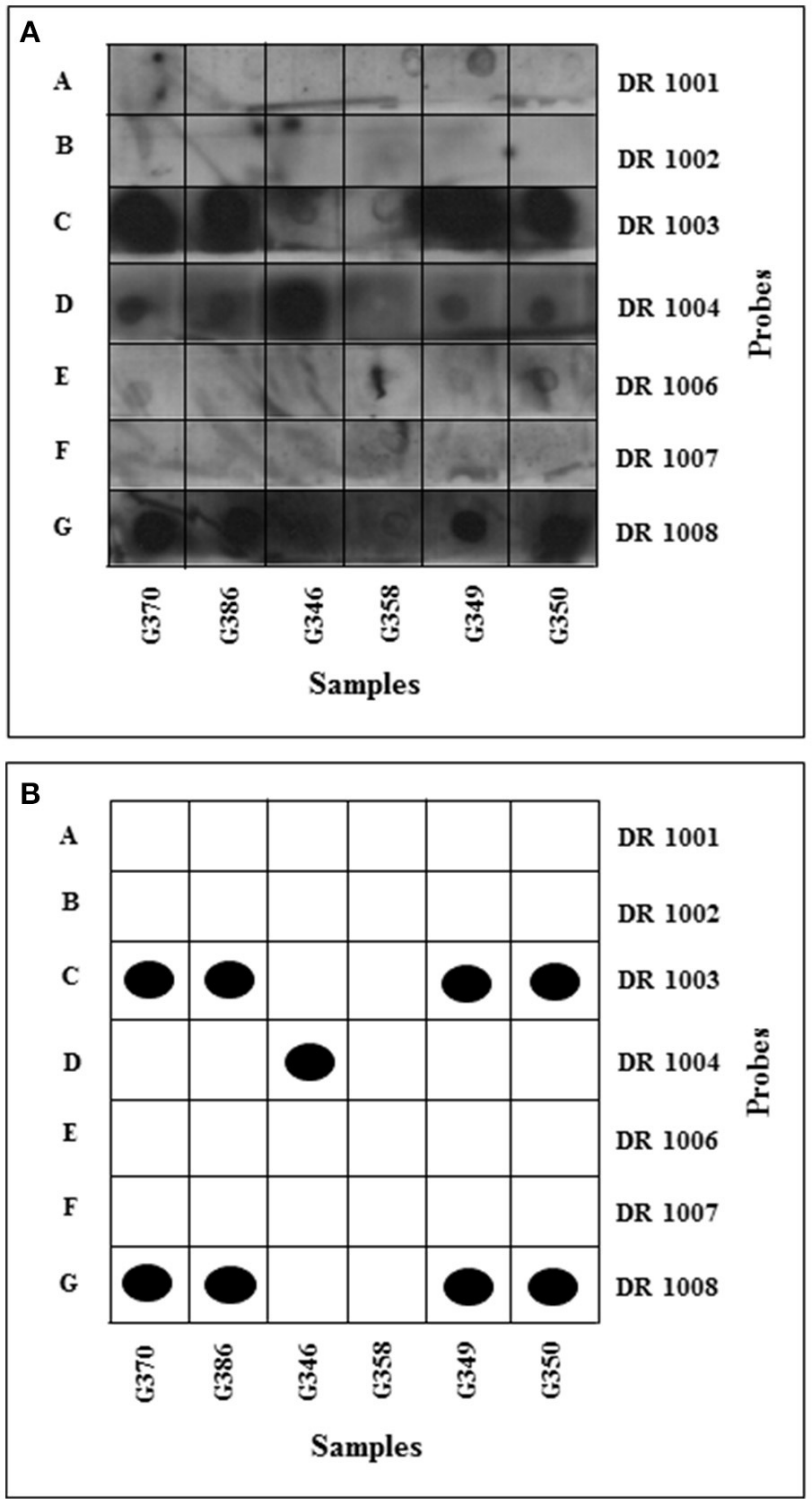
HLA-DRB typing: development of dot blots. The membranes were incubated with luminol and hydrogen peroxide and then exposed to X-ray film for 2 min and developed using an automated developer. **(A)** Samples amplified using DRB1 and 3 primers flanking 1st and 2nd HVR dotted onto positively charged nylon membranes. **(B)** Schematic interpretation of dot blots. Black dots represent unambiguous positive results, with ambiguous results indicated by open boxes. Membrane A hybridized with DR1-specific probe; membrane B hybridized with DR2-specific probe; membrane C hybridized with DR3-, 6-, 11- specific probe; membrane D hybridized with DR4-specific probe; membrane E hybridized with DR7-specific probe; membrane F hybridized with DR9-specific probe; membrane G hybridized with DR10-specific probe.

HLA-DRB typing revealed samples G370, G386, G349, G350 to be heterozygous DR10 and either DR 3, 6, or 11 (Figure 3; Table SI5). DR10 is relatively rare in all populations. To distinguish between DR3, 6, and 11, G370, G386, G349, and G350 being positive for the DR 1003 probe, the four samples were added to the PCR mixture with HLA-DRB primers flanking the third hypervariable region. Three of the four samples (G370, G386, and G349) gave positive PCR results for the third hypervariable region, even though repeated PCR of the first and second regions did not succeed. This could be explained by the flanking distance of the primers and the extent of fragmentation of the templates; primers 1 and 3 (first and second hypervariable regions) gave PCR products of 123-bp whilst primers 4 and 5 resulted in a shorter PCR product of 103-bp. Initially, to split DR 3, 6, and 11, only two probes were used. DR 7004 (DR 3 specific) and DR 5703 (DR 11 specific) were employed on samples G386, G349, and G350. The samples were negative for all probes, suggesting them to be DR6.

Typing of samples G375, G204, G361 and G298 was less successful, as only three out of the seven probes used hybridized to their complementary sequence (Table SI5); as a result only partial types were obtained. Sample G346 was typed as DR4 and possibly DR2, DR7 or DR9. Sample G358 was negative, probably due to the extremely weak PCR product.

Retyping using previous PCR products and PCR products from fresh extracts gave a clearer insight to the types (Table SI5). To obtain more specific serological types, an increased number of probes was used (Table SI7). Despite repeated attempts, probes DR 1002 (DR2 specific), DR 1006 (DR7 specific) and DR 1007 (DR9 specific) did not work. The PCR products generated from the control cell lines were successful, suggesting failure was not due to the controls, but more likely to the probe itself. Retyping of sample G298 and C.P. Voong gave the same result as before. G298 was thus typed as DR1 and possibly DR2, DR7 or DR9, and C.P. Voong as DR4.2.

#### Amplification of the hypervariable segments of the mitochondrial control region

Amplification of extracts from individuals from the vault complex (Figure SI2 and Table SI9) yielded seven samples with complete amplification of the first hypervariable region of the D-loop (G298, G350, G361, G370, G377, G386, G391), and one sample with a partial amplification (G375, first segment), and eight for the second hypervariable region of the D-loop (G298, G350, G358, G361, G370, G377, G375, G386). Samples G370, G298, and G346 were amplified in triplicate using the mitochondrial primers L250 and H390, flanking the second hypervariable region of the D-loop. This revealed the same sequence (Table SI9). There were, however, two variations from the control Anderson sequence: a previously unobserved base substitution (C for T) at position 253, and a C insertion between position 310 and 311, now known as 310.1. The latter has been observed before and is quite common in all ethnic groups (Stoneking et al., 1991).

In an attempt to obtain more positive amplification products, samples G386, G350, and G390 were put through a second round of PCR. This, however, although improving the visibility of the amplification product in some cases, caused primer dimers to form.

Sample G375 was only positive for the first section of the first hypervariable segment of the control region (amplified with primer set A, Table SI6). It was negative for the remaining three. This was despite the fact that the DNA quantitation results suggested that the extract contained sufficient DNA (57 ng/g bone) for the PCR to succeed, even assuming that 95% of the templates were damaged. The use of carrier DNA, more Taq, and other primer sets failed to yield positive PCR results.

The use of more Taq in PEP reactions and routine PCR resulted in the positive amplification of sample G298. With sample G390, concentration of the bone sample extract and doubling the amount of Taq resulted in positive amplification products. Samples from the triple burial (G663, G664, G667) and Group H (G132, G174, G180) were negative.

From the partial sequences obtained, sample G370 was shown to have a base substitution at position 16092 (T for C), as previously observed by Stoneking et al. (1991). Sample G361 was shown to have a substitution from C to T at np 16239 in HVS 1; this variation has been reported before, for example in Poles and Russians (Malyarchuck et al., 2002). Heteroplasmy was observed in sample G390 at position 16246. This was confirmed upon re-sequencing the same region. However, without incorporating the PCR products into cloning vectors and sequencing the recombinant clone, we cannot be absolutely sure of the observed heteroplasmy. Other than G370 and G361, all the samples have a HVR1 identical to the Cambridge Reference Sequence (CRS).

#### Gender determination

Eight samples were positive for gender determining PCR (Figure SI3). The PCR results for samples from the vault complex indicated two males and two females, with three of the four skeletons analyzed giving the same results as those obtained by osteological analysis. For the fourth sample, G298, determined to be female, gender could not be established from osteological characteristics. This agreement between gender identified by the amelogenin sex test and osteology provides some vindication of the integrity of aDNA techniques, providing identification of sex for some of the deceased which was absent before.

## Discussion

In the present study, DNA extracted from 29 Romano-British skeletons, obtained from an urban burial ground at Butt Road, Colchester, was studied with the aim of inferring possible kinship relationships based on the determination of HLA types and sequence polymorphisms of the mtDNA control region.

The HLA-DRB typing results (Figure 3; Table SI5) showed that four samples had the same DRB type (G370, G386, G349, and G350). The four were heterozygous DR10 and DR3/6 or 11. Samples G361 and G298 were positive for DR1. The frequency of DR1 in Britain and Italy is 10.7 and 8.5%, respectively. One of the samples (G346) was positive for DR4 (Figure 3), a type relatively frequent in the British population (13.9%) but less so in Italians (7.1%). The samples were also tested, and shown to be negative for DR8 and DR9 which are rare in European populations.

The prevalence of the HLA-DR10 type is of particular interest as it is present in most populations at a low frequency, 1–2% (Dyer and Middleton, 1993). It is therefore surprising to find a number of samples with this type. Nine of the 10 HLA-DRB typing results were from remains taken from the vault complex, suggesting a possible kinship relationship. However, an ethnographical interpretation of the presence of DR10 is not expedient for the reason that, in the late Roman period, Colchester's population would have included people descended from many places in the Roman Empire. Moreover, none of the individuals in this study was characterized completely at the allele level. Recently, however, a probable case of non-adult thalassaemia has been identified in a 1.0–1.5 year old skeleton from Butt Road (Rohnbogner, 2016). This disease is endemic to the Mediterranean, and cases have provided direct osteological evidence for immigration to Roman England.

Sequencing of samples G370, G298 and G346 revealed the same mitochondrial sequence (Table SI9).There were, however, two variations from the control Anderson sequence. The first is a base substitution (C for T) at position 253 that has not been observed before, according to the Doug Wallace mitochondrial sequence database. The other difference was a C insertion between position 310 and 311, now known as 310.1. This C insertion has been observed before and is quite common in all ethnic groups (Stoneking et al., 1991).

Inferences of kinship relationships could be made only for the hypothesized family groupings in the complex of graves centered on the timber vaults CF55 and G366. Based on stratigraphical information, one of the strongest links was that between graves G350 and G361 (of Group O). These graves, both containing middle-aged males, were precisely set side by side. Both coffins were marked. The HLA-DRB typing results confirmed that both G361 and G350 were positive for the DR 1003 probe, defining DR3, 6, or 11. Subsequent typing narrowed down the types to be possibly DR3 or DR6. DR typing would appear to indicate they share the same HLA haplotype. The suggestion that the remains were those of father and son, or brothers, is thus strengthened.

There is a strong argument for linking G386, the grave of a young adult female, with G362 and G382, containing infants, all of Group P. The graves G386 and G362 were positioned side by side, and G382 lay directly on top of G386. G386 was postulated to be the mother of both infants. DNA extracted from G386 yielded positive mitochondrial and HLA-DRB PCR products. However, G362 and G382 were poorly preserved and found to contain very little, if any, DNA in the extracts.

Grave G386 was also considered possibly related to G370, also in Group P: the two graves were in close proximity to each other and shared the same DR types (DR 10 and DR 3/6). Based on osteological evidence, G370 was the grave of a young adult male. Since G370 and G386 shared the same DR types, the relationship most likely to exist between them is that of brother and sister, as there is a 25% chance of the HLA of siblings being the same. There are, however, discrepancies in this interpretation. Stratigraphical evidence indicated that G386 is much older than G370. The elapsed time between the burials is too great for the supposition to hold, unless the individual buried in G370 was born when that in G386 was into adult life.

Sequencing of part of the second hypervariable segment of the mitochondrial control region indicated that G370 (Group P) and G346 (young female) share the same mitochondrial type, but they had different DR types. Consequently G370 and G346 might also form a brother-sister pair. If, however, assuming that G386 and G370 are brother and sister (based on DR types), the relationship between the two and G346 is likely to be more distant, such as cousins or even uncle/aunt and niece. It was difficult to infer the relationship between G346 and G370 based on grave positions or other archeological evidence, and since the only link between them is that they share the same mitochondrial type, it is thus not a strong one. It is also noted that the hypothesis that G386 and G370 are brother and sister is weakened by the change in position 16092 in G370 (above). This may refute close contemporaneity with G386/G346.

The density of later graves surrounding the vaults CF55 and G366 has been suggested to indicate they form a possible clustering, the vaults being interpreted as containing focal graves, esteemed burials of significance to those buried around them (Crummy et al., 1993). It was proposed that the presence of presumed family burials around the central focus is a typical burial characteristic; the verification of family groupings in the vault complex supports that identification. Focal burials are also a feature of martyrial cemeteries and do not appear in pagan cemeteries of fourth-century Britain. Thus, they have been considered a likely visible Christian trait (Rahtz, 1977; Watts, 1991). Elsewhere in Roman Britain, comparable focal graves have been suggested for Cannington, a fourth- to seventh-century cemetery, in which early family groups have tentatively also been identified (Rahtz, 1977), and Cemetery 3 at Poundbury, dated to the mid fourth century (Watts, 1991). Those at Poundbury involve mausolea, with each the probable focus for family burials (Farwell and Molleson, 1993). However, in the pagan second to third century Trentholme Drive cemetery at York, a pyre, although in disuse, may have been regarded as of special significance for later burials that cluster around it, whether for reasons of cemetery organization or ritual continuity (Hatton, 1999). Potential social and religious meanings for the presence of family graves directly associated with CF55 and G366 are the preservation of family unity and to share in the benefits of propinquity (Watts, 1991).

The presence of family groupings at Butt Road also strengthens the prospect that at least some Period 2 burial plots continued from the Period 1 cemetery. There is considerable evidence that the practices adopted in the Period 2 cemetery were quite different from those in the cemetery that preceded it, most obviously the west-east orientation of the Period 2 inhumations (Crummy et al., 1993). There is also evidence, however, for continuity of some burial plots between Period 1 and 2. For example, five Period 2 graves laid in a row along the line of a ditch form a group remarkably similar to Group C in Period 1 Phase 3, even though they are horizontally but not vertically related (Crummy et al., 1993). Shared characteristics included alignment, close together in neat rows, deposition of CAM 268 pots, and, in one case, footwear, among the Period 2 burials. It is noted that in cemeteries of fourth-century Italy no family member could be denied burial in a private family tomb on the basis of belief (e.g., Christian) unless the head of the family so decreed (Johnson, 1997). A similar practice could have pertained at Butt Road. In this connection, it is interesting that a recent bioarchaeological study of the Butt Road skeletons found little difference in skeletal stress between Period 1 and Period 2 (Jenny, 2011). Haplogroups were assigned using Phylotree data generated by the HaploGrep v.2.0 server (http://haplogrep.uibk.ac.at). The haplogroup classification obtained was based on Kulczynski distance. For each burial specimen and the two controls a lineage graphical representation of the haplogroup classification per sample was generated indicating they are most likely remnants from Roman descent (Supplementary Information).

In summary, the DNA analysis provided corroborative evidence for the existence of family groupings thought to exist in the Period 2 cemetery. Close correspondence between the gender explicit in the DNA on the one hand and the gender apparent through the site evidence and bone on the other was also observed. Additional validation and expansion of these studies, such as analyses of the relevant RFLP of the coding region to verify the HVR1 sequenced to the CRS are worth performing in the future.

## Data availability

The Butt Road skeletal archive is available for additional studies. It is housed at Colchester and Ipswich Museums (CIMS Museum Resource Centre, 14 Ryegate Road, Colchester, Essex CO1 1YG).

Our additional data have been added to the Supplementary information. We have no additional data to include in a public repository.

## Ethics statement

Ethical considerations in this research related only to the requirement of a coroner's license to allow removal and storage of the skeletal remains.

## Author contributions

NF, MW, PRS, and PC conceived the study; CV carried out most of the experimental protocols of the work, and together with PRS participated in the initial data analysis; PSS and NF co-wrote the entire manuscript; EH provided annotated archeological plan drawings; SH and DT participated in data analysis; CN participated in the design of the HLA study and analysis of the molecular data; PC and PSS provided archeological interpretation; PC also contributed to the editing of the manuscript; DT provided molecular genetics support and data analysis.

### Conflict of interest statement

The authors declare that the research was conducted in the absence of any commercial or financial relationships that could be construed as a potential conflict of interest.
