# Supplementary material for: HLA-DR Genotyping and Mitochondrial DNA Analysis Reveal the Presence of Family Burials in a Fourth Century Romano-British Christian Cemetery
Source: Front Genet. 2017 Dec 5;8:182. doi: 10.3389/fgene.2017.00182 (PMC5723391; doi:10.3389/fgene.2017.00182)
Supplement: Supplementary file 1 [file DataSheet1.pdf]

SUPPLEMENTARY INFORMATION (SI)

Supplementary Figures

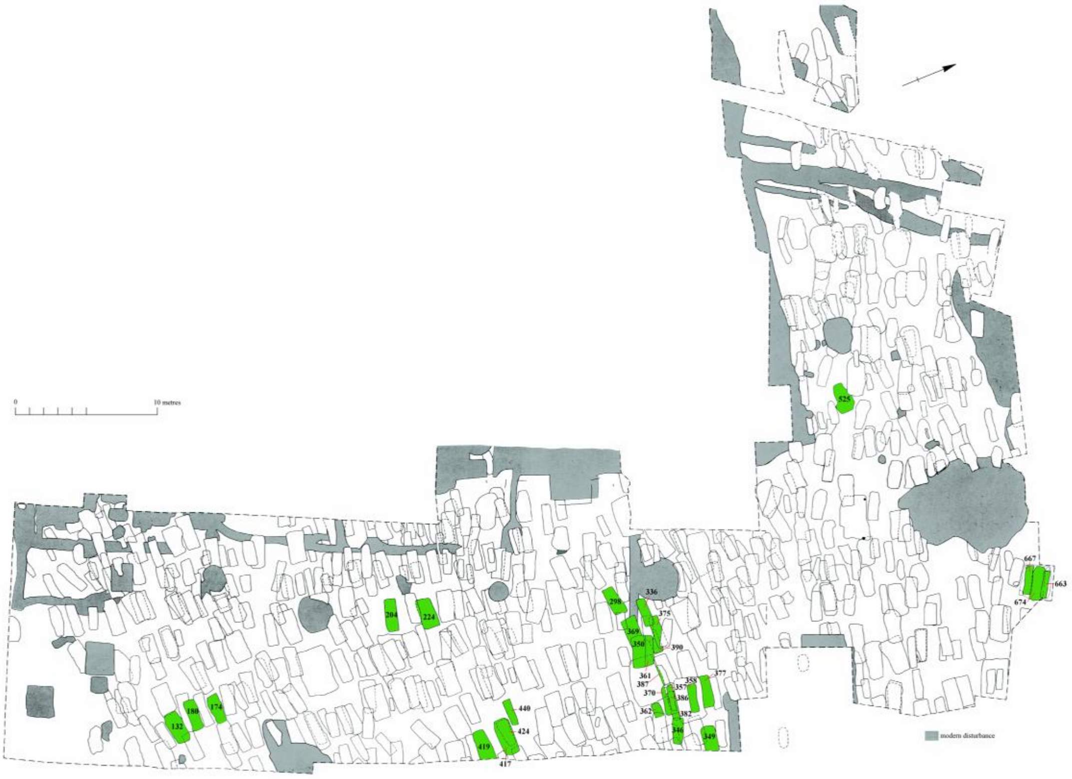

SI Fig.1 The locations of sampled 4th-century graves in the part of the Period 2 cemetery at Butt Road, Colchester, excavated by the Colchester Archaeological Trust.

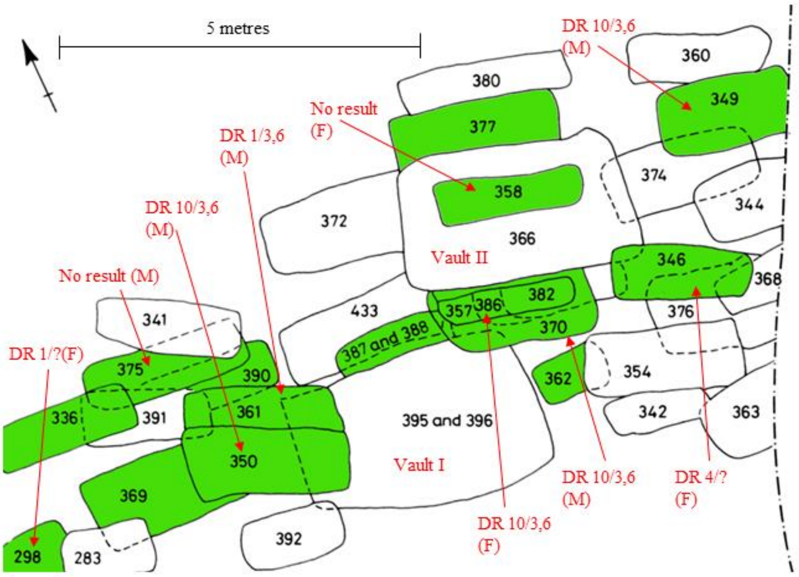

SI Fig. 2 Diagrammatic representation of the vault complex.
The vault complex is formed of graves centred around the timber vaults CF55 and G366. DNA was extracted from skeletal remains from graves marked in green, with HLA-DR typing results shown. M and F denote male and female. Figure adapted from Crummy et al. (1993).

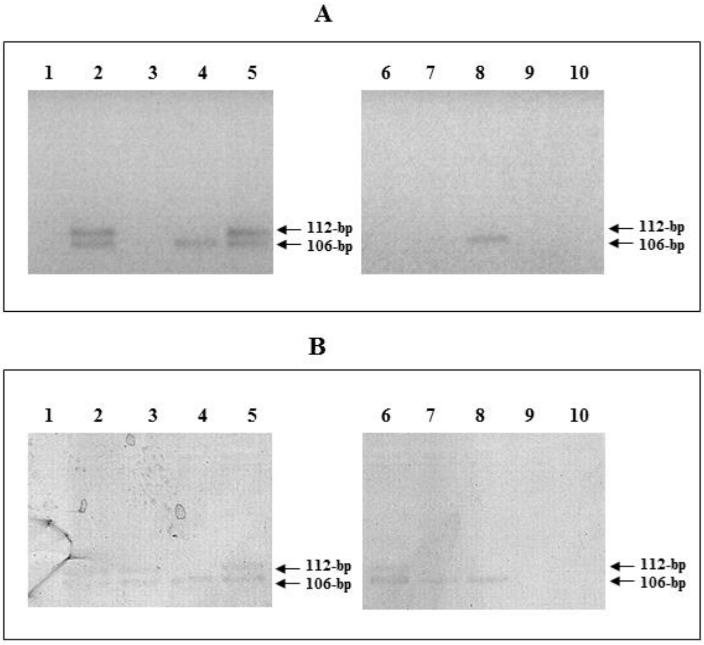

SI Fig.3 Gender determination of samples from the vault complex that had given positive results with HLA-DRB primers.

PCR was carried out using amelogenin primers generating products of 112 and 106 for X and Y homologues respectively. In (A) the PCR annealing temperature used was 60°C. In (B) touchdown PCR was used, the temperature lowered by 1°C for every 5 cycles. The amount of Taq was increased from 1.5 to 3.0 units and the number of cycles from 35 to 40. Lane 1, G386; 2, G361; 3, G375; 4, G346; 5, G370; 6, G350; 7, G358; 8, G298; 9, G349. Lane 10 is the negative control.

Skeletal remains analyzed: possible relationships based on site contexts

In the Butt Road Period 2 cemetery complicated relationships between graves and some coffins were apparent, allowing the inference of direct family relationships. Clear horizontal and vertical stratigraphical relationships were the main criteria in determining possible biological kinship (Crummy et al., 1993). In addition, other possible family burial groups became manifested after the plotting of individuals exhibiting non-metrical traits of the skull. Such less frequent osteological traits most clearly sanction Group R. The archaeological and osteological findings, described below, were put forward by Crummy et al. (1993) to suggest kinship, indicative of family groupings. Samples from all these hypothesized groupings were analyzed.

1. Vault complex (CF55, Vault I, and G366, Vault II)

Several Period 2 burial groups were located within a complex of graves centred around two timber vaults. Vault II contained G366, a single burial of a middle-aged male. Vault I was a double burial containing the coffins of a middle-aged male and female, presumably husband and wife. The interrelationships of graves between the vaults and those surrounding the vaults were well-defined, making it possible to postulate family groupings. The archaeological context of the samples analyzed is as follows (see also SI Fig 1, Fig 2 and Table S4):

Group C: G298, G369, G390 sampled

Group C is a postulated family group comprised of G298, G299, G369, G390 and G433. This group is first identified in Period 1 Phase 3. Two Group C graves (G298, G390) were placed in the group by virtue of their grave goods and two more (G369, G433) owing to their position relative to the first two. In addition, G295 and G320 may also belong to Group C based on their deposits. However, their alignments slightly differ.

The three Group C graves G299, G298 and G369 lay head to foot in a row which corresponded roughly to the line of CF59 projected westwards towards CF32. The two sampled Group C graves, G369 (young adult female) and G298 (adult, sex uncertain), cut G283 (neonate), and thus either may be related to that burial. It is also possible that G283 is related to G285 (male, age uncertain) which lay adjacent to it in the south, or to G392 (neonate), just over 1 m to the east.

Group O: G350, G358, G361 sampled

Group O in the vault complex comprises G341, G350, G361, and possibly G354 and G358, linked by markers and alignment. A clear relationship exists between G361 and G350, belonging to different stratigraphical levels, Section 5 and 6 respectively. Both G361 and G350 were middle-aged males. Their graves were precisely set side by side. The top of both coffins was marked. Grave G358 was set directly over the G366 vault, suggesting a direct relationship between them.

Group P: G387, G388; possible stacked burial G386/G382/G370/G357*; G362 (samples: G357, G362, G370, G382, G386, G388 sampled)*

Immediately to the south of G366 was the grave of a young female adult (G386), with that of an 18-month to two-year-old child (G382) set into its fill. A close relationship may also be inferred between G386 and G382 with the graves of two newborn infants, G387 and G388, in Section 2 (Group P), linked by alignment and possibly age. Both burials may be related to G433, into which the graves were cut, or to G386, which lay end to end with them. Another grave possibly related to G386 is G362 (neonate), a Section 3 burial. Thus the graves G362, G387, G388, G382 and G386 may potentially be of a mother with four of her children. Grave 370, a young adult male, lies above G386 and G382, and may be associated with them.

Grave 357 directly overlies G370 as the final burial of a possible stack of four related burials between the two vaults, G386/G382/G370/G357. Deliberate stacking, rather than the accidental setting of grave above grave, is perhaps implied, in that G370 lies directly above G386 and G382. Alternatively, G357 and G358 may be more directly related to each other rather than to the underlying burials.

In addition to the above, it is also possible to argue that G370 and G377 (young adult female) may have been a pair, since the alignment of the two graves was similar and they may have originally been side by side. The later vault G366 was dug between the two (SI Fig 2).

Sequence of four graves: G346, G358, G366, G377 (samples: G346, G377sampled)

Graves G346 (young adult female) and G377 (young female) are part of a sequence of four graves, the others being G358 and G366. Grave 377 is second in the sequence, with three graves post-dating it (G346, G358, G366).

Grave 336:

The grave of a young adult male.

Grave 349:

The graves G349 and G344, together with G368 (from Section 5) may have formed a family grouping. The three were well aligned in an evenly spaced row (SI Fig 2).

Grave 375:

The grave of a middle-aged male.

2. Vault 225

Grave 204:

Isolated grave (young adult male), located in the area immediately west of the vault G225. The absence of nearby burials was probably due to Plot B's late incorporation into the Period 2 cemetery.

Grave 224:

The grave of a middle-aged female, lying north-west of timber vault G225.

3. Postulated Period 2 family groupings not associated with vaults

Group H: G132, G171, G174, G180 (samples: G132, G174, G180 sampled)

Group H is located in the south east of the excavation. The three graves, G132, G174 and G180, contained a highly characteristic deposit of glass tableware. Grave 174 also contained jewelry, which provides a link to the fourth member of the group, G171, the only non-adult grave in the group and the only one which was not accompanied by a glass vessel. Together, these graves, linked also by alignment (the four graves lie in a row), form a well-defined group.

Group R: G417, G419, G440 (all sampled)

Group R, positioned near the centre of the eastern boundary of the Period 2 cemetery, is well-defined. It consists of three individuals possessed of a common non-metrical variant of the skull, an inca bone. The graves, two final and one isolated, are assumed from stratigraphy to be a family, being placed close together without undercutting, regularly spaced, and similarly aligned.

Triple burial: G663, G667, G674 (all sampled)

The grouping G663/G667/G674 is interpreted as a multiple burial, as corroborated by clear direct stratigraphical relationships, which also suggest possible family relationships. The three burials were deposited within a large isolated Period 2 grave pit, HF53, in which G663 was the latest burial, emphasizing their association.

4. Period 2 grave (see SI Fig 1)

Grave 525

The grave of an elderly male. Isolated burial.

5. Unnumbered femur

A femur excavated during the study from a Roman site at Colchester was also analyzed. The bone was dug out from the ground and placed into an UV irradiated autoclave bag. Only C.P. Voong had contact with the femur. Therefore the only opportunity and source of contaminating the sample with contemporary human DNA would be from him. This made the process of authenticating subsequent DNA sequences less complicated.

Materials and methods

1. The efficiency of the silica-based extraction methodology

The silica-based extraction technique involved vigorous washing of the silica pellet, causing some DNA loss. To investigate the efficiency of this method and to determine the amount of DNA recovered, known amounts of DNA (0.2 -1.0 µg of 1kb ladder) were added to the DNA extractions. A known quantity (10 µg) of the 1kb molecular weight ladder DNA was added to 2 ml of extraction buffer and incubated at 50°C for 1 h in an orbital shaker. A negative control, containing everything except the DNA, was performed in parallel. The tubes were then centrifuged for 10 min at 12,000g. The supernatant was transferred to 1.5 ml screw on cap micro-centrifuge tubes with 30 µl of silica suspension and incubated for a further 45 min at room temperature on a tube rotator. PicoGreen was used to determine how much DNA was recovered in each extraction.

2. Comparing decalcified and non-decalcified samples

Initially, 10 bone samples (G204, G174, G346, G350, G358, G370, G417, G390, G361, G298) were decalcified before DNA extraction. Powdered bone (1 g) from each sample was decalcified with 50 volumes of 0.5M EDTA, pH 8.5, and incubated at room temperature on a tube rotator for 72 h with two changes of EDTA. However, negative samples seen on the gel were possibly related to the decalcification, which can result in DNA loss. To assess the effect of decalcification on DNA yield the 10 samples were re-extracted with the decalcification step omitted. The DNA extracts were again run on an agarose gel and used for ultraviolet (UV) fluorescence analysis.

The effect of decalcification on DNA loss was also determined through a UV analysis using the dye PicoGreen. Sample G375 was selected, as this sample was well preserved and therefore more likely to contain DNA. The sample was decalcified and extracted as above, the EDTA supernatant was retained and the DNA eluted once with 150 µl Purite water. A 100 µl aliquot of the DNA extract, EDTA supernatant and EDTA was added to a PicoGreen working solution and the fluorescence taken between 500-600 nm.

The agarose gel revealed smearing that was concentrated in the high molecular weight range. The smearing was more evident in non-decalcified samples than in the decalcified samples. Thus omitting the decalcification step does appear to make some improvement. However, the concentration of the smearing in the high molecular weight range suggests it may not be caused by authentic aDNA. Ancient DNA is heavily degraded, thus smearing would be expected lower down (approx. 100-200 bp). The fluorescence could be caused by Maillard products of reducing sugars. These contaminants are known to elute with aDNA and the resulting extracts exhibit a blue/green fluorescence under UV light. However the fluorescence observed was not blue/green, but white. Although the agarose gels indicate that decalcification does make a difference, it is only slight.

The UV absorbance results for decalcified and non-decalcified samples were very similar. The purity of the DNA extracts, judging from the 260/280 nm values, again indicated that the extracts were not pure and contained microbial contaminants. Therefore, this was not an accurate estimation for aDNA concentration.

The DNA extract from sample G375, visualized using the PicoGreen fluorescence assay, gave a peak fluorescence intensity of 103 nm (unadjusted). The EDTA supernatant exhibited extremely high fluorescence (over 300 nm) compared to the DNA extract, making it questionable whether the fluorescence was due to the EDTA itself or DNA. EDTA, however, does not bind to PicoGreen.

3. Investigating the loss of DNA through decalcification using enzymatic hydrolysis

To confirm whether the fluorescence intensity observed in the EDTA supernatant was caused by DNA lost during decalcification or was due to some other component which also binds to PicoGreen, a nuclease was used. The rationale behind the use of a nuclease was that the enzyme would hydrolyze the DNA molecule, rendering it incapable of binding to the PicoGreen dye, so resulting in a decrease of fluorescence intensity. The hydrolysis occurs at all phosphate linkages in the DNA backbone but prefers those between purine and pyrimidine bases. The P1 nuclease was chosen to hydrolyze the DNA.

A control experiment was used to observe whether the fluorescence intensity would be reduced as a result of cleaving the DNA. 100 ng of Lambda DNA was pipetted into a micro-centrifuge tube (MCC) with 16 units of the P1 nuclease added. In a separate MCC tube, 100 ng of Lambda DNA was added with no P1 nuclease added. To observe whether EDTA inhibits the enzymatic activity of P1 nuclease, EDTA with 100 ng of Lambda DNA with and without P1 nuclease was prepared in parallel. The tubes were incubated in a 37°C water bath for 10 min. After incubation the mixtures were added to a working solution of PicoGreen and the fluorescence taken between 500-600 nm.

The hydrolysis of the Lambda DNA caused the fluorescence intensity to fall. The spectra for EDTA mixed with DNA with and without P1 nuclease added showed virtually no difference in fluorescence intensity. It would appear the 0.5M EDTA inhibits the enzymatic activity of the P1 nuclease.

4. EDTA enhances the fluorescence of DNA

EDTA also appears to enhance the fluorescence of DNA. When the fluorescence was read, the sample with no EDTA gave a peak fluorescence intensity of 335 nm compared to 700 nm for the EDTA. As EDTA itself exhibited only background fluorescence, we can conclude that EDTA does enhance the ability of the DNA to intercalate PicoGreen. From this observation, it is possible that the high fluorescence observed in the EDTA supernatant could be due to DNA lost during decalcification.

5. Fluorescence assay of DNA in calcified and decalcified bone samples

The purpose of using P1 nuclease was to determine whether the high fluorescence observed in the EDTA supernatant was due to DNA. However, since EDTA inhibits the activity of the enzyme, it was not possible to determine directly whether DNA was present in the EDTA supernatant. The only option left was to compare decalcified and non-decalcified extracts using the fluorescence assay. Sample G375 was divided into two sets, each of 2 g of bone powder. One set was decalcified and the other set left non-decalcified. The DNA was eluted once in 2 x 150 µl of Purite water. Both extracts were added to a working solution of PicoGreen and spectra taken between 500-600 nm.

To demonstrate that it was DNA that caused the fluorescence in the extracts, the P1 nuclease was added to an aliquot of the non-decalcified sample. After incubation at 37°C for 10 min, the extract was mixed with PicoGreen and the fluorescence taken.

The mass spectrum showed that peak fluorescence intensity was lower for the decalcified sample (110 nm) than for the non-decalcified sample (165 nm). This indicates DNA is lost through decalcification. The spectra showed the fluorescence was reduced compared to the extract not treated with nuclease. Although the use of nuclease has not allowed us to determine directly whether DNA caused the extremely high fluorescence observed in the EDTA supernatant or not, it has allowed us to establish beyond doubt that aDNA is being recovered, since as much as half of the DNA appears to be lost through decalcification. The amount of DNA lost during decalcification was not determined for every femur sampled. However, sample G375 clearly demonstrates that decalcification does result in the loss of DNA. Therefore decalcification for subsequent extractions was omitted.

6. Effectiveness of removing collagen from the extract

Collagen has been identified as a major inhibitor of PCR. This protein constitutes around 90% of the organic fraction of bone (1/3 by weight), the remaining 10% (2/3 by weight) consisting mainly of water and inorganic material, most of which is calcium phosphate. To investigate the effectiveness of removing collagen from the extract, bone powder from the same sample was extracted with the addition of the protease collagenase A (Boehringer Mannheim). The collagenase preparation used was neither pure nor sterile and is frequently contaminated with other proteases. Sterile collagenase, guaranteed to be free of DNA, was not available from any supplier. To monitor possible contaminating DNA originating from the collagenase preparation, stringent extraction controls were performed in parallel with the sample.

After collagenase treatment, the extract was eluted once with 150 μl Purite water. The modified extract was added to PEP-PCR with 0.2, 0.6, 1.0, 1.25 and 1.5 μl of Taq. An aliquot of the PEP-PCR products was added to specific mitochondrial PCR with primers L16055 and H16142 flanking a 125-bp region of hypervariable segment I of the mitochondrial control region. Both PEP and PCR products were mixed with loading buffer and electrophoresed through a 3% agarose gel pre-stained with ethidium bromide. The extract was also used to spike control PCR.

Removing collagen through enzymatic hydrolysis greatly reduced inhibition. However, inhibition could not be fully prevented. This suggested that it was not inhibitors that were disrupting amplification, but rather that it was the damaged and modified aDNA that did not allow amplification. This, however, was found not to be the case, since small volumes (μl) of aDNA extracts added to PCR mixture with abundant undamaged templates were shown to inhibit and reduce amplification. Through modification of the standard PCR, including the use of wax-mediated hot start and touchdown PCR, specific amplification products of low target copy templates were obtained. By diluting the extracts, using more Taq and adding BSA to the PCR mixture, inhibition of the PCR was generally overcome. Despite the positive PCR, amplification was still hindered compared to the control (with no ancient extracts added) in which amplification resulted in an extremely bright band. The control PCR reaction with no inhibition indicated that amplification was achieved with 2 units of Taq.

6.1 Standard and modified extracts

As a further control, standard and modified extracts (5-15 µl) were added to the control PCR mixture with mitochondrial primers. Amplification was observed in all PCR reactions spiked with the modified extracts. However, for PCR reactions spiked with standard extracts, amplification, despite being extremely weak, was detected for the reaction with 5µl of ancient extract added but negative for 10 and 15 µl. The template used for the control PCR reaction was from Raji cells, extracted with the same method as that used for extracting DNA from the bones.

7. Determining the amount of Taq required to overcome inhibitors

Inhibition of the PCR can be overcome by increasing the amount of Taq. To determine how much Taq is required to overcome the inhibitors, aliquots of the extracts (first and second elution) were added to the PEP-PCR mixture with 5, 10 and 15 units of Taq. Aliquots (10 µl) of the PEP PCR product were added to mitochondrial PCR with primers L250 and H390 flanking a 140 bp region of hypervariable region II of the mitochondrial genome. Another aliquot of the PEP product was run on a 2.5% agarose gel.

PEP-PCR of the first elution extract showed that PEP was negative for 5 and 10 units and positive for 15 units of Taq, whereas for the second elution extract PEP was negative for 5 units but positive for 10 and 15 units of Taq. Subsequent specific mitochondrial PCR with 5 units of Taq showed that all positive PEP-PCR samples were positive and negative PEP-PCR samples were negative.

8. HLA-DRB typing using PCR-SSOP

In PCR-SSOP typing, the PCR product is fixed on a nylon membrane and the probes are then added. HLA-DRB PCR products (3µl), amplified using HLA-DRB primers, were dotted onto positively charged nylon membranes and left to dry at room temperature for 15 min. The double-stranded PCR products were denatured by submerging the membrane into denaturing solution (1.5M NaCl, 0.5M NaOH) for 5 min. The denaturing solution was neutralized by submerging the membrane into neutralizing solution (1.5M NaCl, 0.5M Tris-HCl pH 7.4) for 1 min. The membranes were dried for 10 min at 80°C. To ensure the DNA was securely attached to the membrane, the membranes were exposed to UV light for 1 min.

The membranes were placed into 50 ml centrifuge tubes containing 10 ml of pre-hybridization buffer [1x NaCl, 1x sodium citrate (SSC), 0.002% SDS, 1% blocking agent (milk powder), 0.1% N-laurylsarcosine]. They were incubated for 1 h at 42°C, with continuous shaking in a hybridizer (Techne Cyclogene).

The membranes were first washed (non-stringent 5x SSC, 0.1% SDS) at room temperature for 5 min, and then washed (stringent 1x SSC, 0.1% SDS) at a specific temperature for 30 min. After the stringent wash, the nylon membranes were washed twice in buffer (1.0M Urea, 0.1M NaCl, 5% Triton X-100, 1% Dextran Sulphate) for 1 min at room temperature before being submerged in 25 ml of the same buffer containing horse radish peroxidase (HRP).

Hybridization of the probe with the amplified DNA was detected using chemiluminescence. Equal volumes of the detection reagents containing luminol and H_2O_2 were mixed and applied to the membrane. After incubation for 2 min, the membranes were wrapped in Saran

Wrap. The exposure of the detection reagents to HRP produces a blue light that is detected on X-ray film. The membranes were exposed to X-ray film for 5 min. After removing the first film, a second exposure was taken immediately; the exposure time for second was dependent on the signal intensity obtained with the first.

Archaeological context	*Grave number*	*Sex*	*Age*	*Preservation A*	*Preservation B*	*DNA recovered*
Vault complex (burials surrounding CF55, Vault I, and G366, Vault II)						
Group C						
	G298	?	Adult	Poor	***	Yes
	G369	F	Young	Poor	**	No
	G390	?	Adult	Poor	***	Yes
Group O						
	G350	M	Middle-aged	Good	***	Yes
	G358	F	Young	Good	***	Yes
	G361	M	Middle-aged	Good	****	Yes
Group P						
	G357	M	Middle-aged	Poor	*	No
	G362	I	Infant	Poor	*	Yes
	G370	M	Young	Good	****	Yes
	G382	I	Infant	Poor	*	Yes
	G386	F	Young	Good	****	Yes
	G388	I	Infant	Poor	*	Yes
Sequence of four graves (G377, G346, G358, G366)						
	G346	F	Young	Good	****	Yes
	G377	F	Young	Good	***	Yes
Grave 336	G336	M	Young	Poor	**	Yes
Grave 349	G349	M	Middle-aged	Good	****	Yes
Grave 375	G375	M	Middle-aged	Good	*****	Yes
Vault (G225)						
Grave 204	G204	M	Young	Good	*****	Yes
Grave 224	G224	F	Middle-aged	Good	****	Yes
Group H						
	G132	?F	Middle-aged	Poor	***	Yes
	G174	F	Adult	Poor	***	Yes
	G180	M	Middle-	Good	****	Yes

			aged			
Group R						
	G417	F	Young	Poor	***	Yes
	G419	?	Middle-aged	Poor	**	Yes
	G440	F	Middle-aged	Poor	**	Yes
Triple burial						
	G663	?	Old	Poor	**	Yes
	G667	I	17-18 yrs	Poor	*	Yes
	G674	?	Old	Poor	**	Yes
Period 2 grave						
	G525	M	Old	Poor	**	No

SI4 Table. Details of skeletal remains provided by the Colchester Archaeological Trust for DNA analysis. All the samples belong to Period 2. F, M, I denote male, female or indeterminate, whereas a question mark before F or M denotes probable female or male. Preservation of the whole skeleton (Preservation A) is described as either good or poor. Preservation of individual femurs (Preservation B) is indicated by the number of asterisks, from one (poor) to five (exceptional). Groups of Period 2 graves identified by Crummy et al. (1993) are indicated.

Sample	DRB type (1)	DRB type (2)	DRB type (3)
G370	DR 10/3,6,11		DR 10/3,6
G386	DR 10/3,6,11		DR 10/3,6
G346	DR 4		
G358	No result		
G349	DR 10/3,6,11		DR 10/3,6
G350	DR 10/3,6,11		
G375	No result	No result*	
G204	DR 3,6,11/?	DR 3,6,11/3,4*	DR 3/3,6
G298	DR 1/?	DR 1/2,7 or 9	
G361	DR 3,6,11/1	DR 3,6,11/1*	DR 3,6/1
Research worker	DR 4	DR 4/2, 7 or 9	

SI5 Table. Summary of HLA-DRB typing results. (1). First round typing. (2). Retyping of first round samples. (3). Final DR type after splitting DR 3/6 and 11 using third hypervariable region. Asterisks indicate typing from fresh PCR product.

	Primers	*Sequence (5'-3')*	*% GC*	*Tm (°C)*	*Ta (°C)*	*Ta (°C) used*
A	L16055	GAA GCA GAT TTG GGT ACC AC	50	60	55	*53*
	H16142	ATG TAC TAC AGG TGG TCA AG	45	58	53	
B	L16131	CAC CAT GAA TAT TGT TCA AG	45	56	51	51
	H16218	TGT GTG ATA GTT GAG GGT TG	50	58	53	
C	L16209	CCC CAT GCT TAG AAG CAA GT	50	60	55	*52*
	H16303	TGG CTT TAT GTA CTA TGT AC	35	54	49	
D	L16287	CAC TAG GAT ACC AAC AAA CC	45	58	53	55
	H16379	CAA GGG ACC CCT ATC TGA GG	60	64	59	
E	L250	TGT CTG CAC AGC CAC TTT CC	55	62	57	55
	H390	TCT GGT TAG GCT GGT GTT AG	50	60	55	
F	HLA 1	CAC CCG ACC ACG TTT CTT	55	61	51	
	HLA 2	CCG CAC CCG CTC CGT CCC	83.3	66	61	
G	HLA 1	CAC CCG ACC ACG TTT CTT	55	61	51	
	HLA 3	GTC GCT GTC GAA GCG CAC	66.7	61	55	
H	HLA 4	GTG ACG GAG GTG GGG CGG	77.8	64	59	
	HLA 5	CCC GTA GTT GTG TCT GCA	55.6	56	51	

SI6 Table. A to D. Mitochondrial primers. Primer sets A-D flank overlapping fragments of the first hypervariable segment generating products of 125, 125, 132 and 130 base pairs, respectively. Primer set E flanks part of the second hypervariable segment, generating a product of 140 base pairs. F to H. HLA-DRB primers. F. HLA-DRB primers flanking first hypervariable region of the second exon. G. HLA primers 1 and 3 flanking first and second hypervariable region of the second exon of HLA-DRB. H. Primers flanking third hypervariable region of HLA-DRB.

Probe	*Sequence (5'-3')*	*Ta (°C)*	*Specificity*
DR 1001	GTGGCAGCTTAAGTTGA	47	DR 1
DR 1002	GCT AAG AGG GAG TGT CAT	49	DR 2
DR 1003	GTACTCTACGTCTGAGTG	49	DR 3,6,11
DRI004	GAGCAGGTTAAACATGAG	47	DR4
DR 1006	TGG GAG GGT AAG TAT AAG	47	DR 7
DR1007	GAAGCAGGATAAGTTTGA	45	DR9
DR1008	GAGCAGGTT,AAGTTTGAG	47	DR 10
DR 3703	AG GAG GAG GAGMC GTGG	53	DR 3,6
DR 7004*	GGC CGG GTG GAG AAG TAG	53	DR 3
DR 5703*	GGCTGATGAGGAGTAGTG	51	DR 11
DR 5704*	G CCT GGT GCG GAG GAG TG	53	DR 14
DR ALL*	TGG MC AGC GAG AAG GAG	51	ALL DRB

SI7 Table. Sequence-specific oligonucleotide probes used for HLA-DRB typing. Probes marked with an asterisk were used on the third hypervariable region to split DR3, 6 and 11. Ta denotes the annealing temperature used for hybridization.

Archaeological Context	Grave number	Sex	1st segment HVR1	2nd segment HVR1	3rd segment HVR1	4th segment HVR1	HVR2	HLA-DR	Gender Determination
Vault complex (burials surrounding CF55, Vault I, and G366, Vault II)									
Group C									
	G298	?	positive	positive	positive	positive	positive	positive	positive
	G369	F	negative	negative	negative	negative	negative	negative	negative
	G390	?	positive	positive	positive	positive	negative	negative	negative
Group O									
	G350	M	positive	positive	positive	positive	positive	positive	positive
	G358	F	negative	negative	negative	negative	positive	positive	positive
	G361	M	positive	positive	positive	positive	positive	positive	positive
Group P									
	G357	M	negative	negative	negative	negative	N/A	negative	N/A
	G362	I	negative	negative	negative	negative	negative	N/A	N/A
	G370	M	positive	positive	positive	positive	positive	positive	positive
	G382	I	negative	negative	negative	negative	negative	N/A	N/A
	G386	F	positive	positive	positive	positive	positive	positive	negative
	G388	I	negative	negative	negative	negative	negative	N/A	N/A
Sequence of four graves (G377, G346, G358, G366)									
	G346	F	negative	positive	negative	positive	positive	positive	positive
	G377	F	positive	positive	positive	positive	N/A	negative	negative
Grave 336	G336	M	negative	negative	negative	negative	N/A	negative	negative

Grave 349	G349	M	negative	negative	negative	negative	positive	positive	negative
Grave 375	G375	M	positive	negative	negative	negative	N/A	positive	positive
Vault (G225)									
Grave 204	G204	M	positive	positive	positive	positive	positive	positive	positive
Grave 224	G224	F	negative	negative	negative	negative	negative	negative	N/A
Group H									
	G132	?F	negative	negative	negative	negative	N/A	negative	N/A
	G174	F	negative	negative	negative	negative	positive	negative	N/A
	G180	M	negative	negative	negative	negative	N/A	negative	N/A
Group R									
	G417	F	negative	negative	negative	negative	negative	negative	N/A
	G419	?	negative	negative	negative	negative	N/A	negative	N/A
	G440	F	negative	negative	negative	negative	N/A	negative	N/A
Triple burial									
	G663	?	negative	negative	negative	negative	N/A	negative	N/A
	G667	I	negative	negative	negative	negative	N/A	negative	N/A
	G674	?	negative	negative	negative	negative	positive	negative	N/A
Period 2 grave									
	G525	M	negative	negative	negative	negative	negative	N/A	N/A

SI8 Table. Mitochondrial, HLA-DRB, and gender determining PCR results. The amplification products encompassing the first mitochondrial hypervariable region (HVR1) were obtained for eight samples, and a partial PCR success for a further sample. To obtain the sequence encompassing HVR1, four overlapping fragments were required. A single pair of primers was used to amplify the second hypervariable region (HVR2). Ten samples were positive for HLA-DRB and eight for gender determining PCR. Groups of Period 2 graves identified by Crummy et al. (1993) are indicated. Abbreviation: N/A, not attempted.

SAMPLES		253		309.1		310.1	
CONTROL	G T	C	T G C A C A G C C A C T T T C C A C A C A G A C A T C A T A A C A A A A A A T T T C C A C C A A A C C C C C C C	-	T	-	C C
G346		T				C	
G346		T				C	
G346		T				C	
G370		T				C	
G370		T				C	
G370		T				C	
G298		T				C	
G298		T				C	
G298		T				C	
RESEARCH WORKER				C		C	

SI9 Table. Mitochondrial DNA sequences from the second hypervariable segment of the control region. Positions different from the Anderson (control) sequence are numbered. The polymorphism at position 253 has yet to be reported in the modern population. The 'research worker' (C.P. Voong) differs from the samples and at position 253 and 309.1.

Phylotree generation

Methods

The mtDNA sequencing data and SNP data were used to generate proper HSD files using the mtDNAProfiler server (http://mtprofiler.yonsei.ac.kr/). The haplogroups were automatically assigned using Phylotree data by the HaploGrep v.2.0 server (http://haplogrep.uibk.ac.at). Haplogroup classification was based on Kulczynski distance. For each burial specimen and the two controls a lineage graphical representation of the haplogroup classification per sample was generated.

Legend:

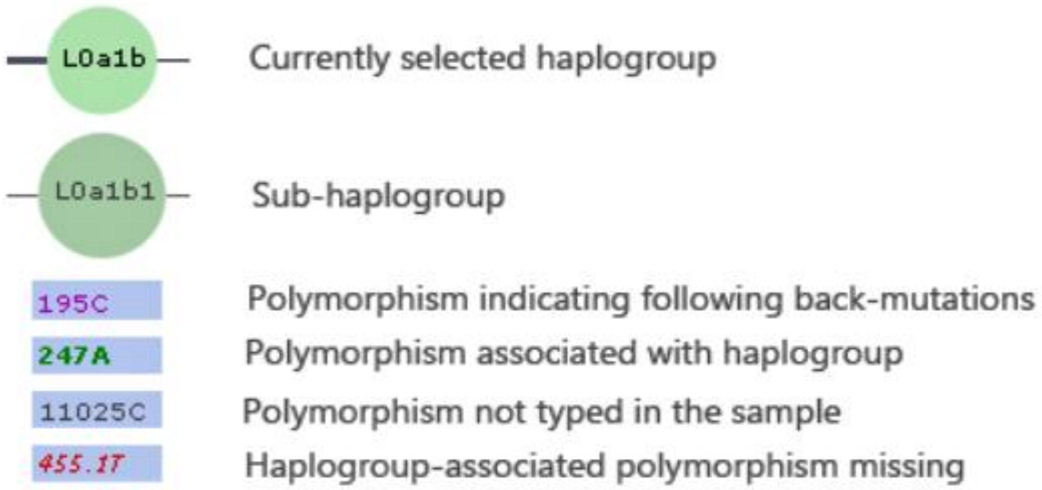

Description of the ethnical distribution of the haplogroups

Haplogroup H2a2a1 (Control, G370): The H2 haplogroup is common in Eastern Europe and the Caucasus:

Haplogroup J2a1a1a (G298, G346): Haplogroup J occurs in approximately 12% of native European populations, with a >2% frequency distribution of mtDNA J within Europe. Subgroup J2a is homogenously spread in Europe, but absent in the nations around the Caucasus. It is not known to be found elsewhere. The time of origin of the younger branches of mtHG J for J2a1a1a is 7,591.6 ± 2,889.6 (between 4,700 and 10,500 before present (ybp).

Haplogroup H1+16239 (G361): H1 encompasses an important fraction of Western European mtDNA lineages, reaching its local peak among contemporary Basques (27.8%). It also occurs at high frequencies elsewhere in the Iberian Peninsula, as well as in the Maghreb (Tamazgha). The haplogroup frequency is above 10% in many other parts of Europe (France, Sardinia, parts of the British Isles, Alps, large portions of Eastern Europe.

Results

The haplogroup H2a2a1 is the most common and is represented by the Control mtDNA and in specimens from G370. It is a subtype of the “Helena” clan haplogroup (Sykes, 2007: "Saxons, Vikings and Celts: The Genetic Roots of Britain and Ireland"). H2a2 is found throughout Europe (ref: https://www.eupedia.com/europe/Haplogroup_H_mtDNA.shtml), but in England it is most prevalent in Wales (up to 60%) (Ref: https://www.eupedia.com/genetics/britain_ireland_dna.shtml).

The haplogroup H1+16239 are represented in specimens from G361. It is present mostly in Scotland and Ireland, but is absent in Wales (ref: https://www.eupedia.com/genetics/britain_ireland_dna.shtml). H1 is by far the most common subclade in Europe, constituting more than half the H lineages in Western Europe (ref: https://mbe.oxfordjournals.org/content/24/2/436.abstract), with the highest frequencies of H1 observed in the Iberian peninsula, south-west France and Sardinia, suggesting possible Roman descent of the burials.

The haplogroup J2a1a1a, represented in the specimens from G298 and G346, is most prevalent in Wales, Scotland and Ireland (https://www.eupedia.com/genetics/britain_ireland_dna.shtml), with J2a1 being found mostly in western, central and northern Europe, particularly around the Alps. (https://www.eupedia.com/europe/Haplogroup_J_mtDNA.shtml). The J haplogroup subtype of the "Jasmine" clan gave rise to the ramification J2a1a1a that branched from J2 around 7,591.6 ± 2,889.6 ybp (between 4,700 and 10,500 ybp).

Sample ID	Range	Haplogroup	Cluster	Overall Rank	Not Found Polys	Found Polys	Remaining Polys	Input Sample
**MTDNA_CONTROL**	16024-16569 ; 251-312 ;	H2a2a1	[H2a2a1, H2a2a1a, H2a2a1b, H2a2a1e, H2a2a1f, H2a2a2]	0.5000				
**MTDNA_G298**	16024-16569 ; 251-313 ;	J2a1a1a	[J2a1a1a, J2a1a1a2, J2a1a1a2a, J2a1a1a3]	0.5893	263G 295T 16069T 16126C 16145A 16231C 16261T	310.1T	253T	253T 310.1C
**MTDNA_G346**	16024-16569 ; 251-313 ;	J2a1a1a	[J2a1a1a, J2a1a1a2, J2a1a1a2a, J2a1a1a3]	0.5893	263G 295T 16069T 16126C 16145A 16231C 16261T	310.1T	253T	253T 310.1C
**MTDNA_G361**	16024-16569 ; 251-313 ;	H1+16239	[H1+16239, H1bf, H1bg, H1bh, H1ch]	0.6835	263G	16239T		16239T
**MTDNA_G370**	16024-16569 ; 251-313 ;	H2a2a1	[H2a2a1, H2a2a1a, H2a2a1b, H2a2a1e, H2a2a1f, H2a2a2]	0.5000			253T 310.1C 16092C	253T 310.1C 16092C
**MTDNA_RESERACH_WORKER**	16024-16569 ; 251-313 ;	J2a1a1a	[J2a1a1a, J2a1a1a2, J2a1a1a2a, J2a1a1a3]	0.5893	263G 295T 16069T 16126C 16145A 16231C 16261T	310.1T	309.1C	309.1C 310.1C

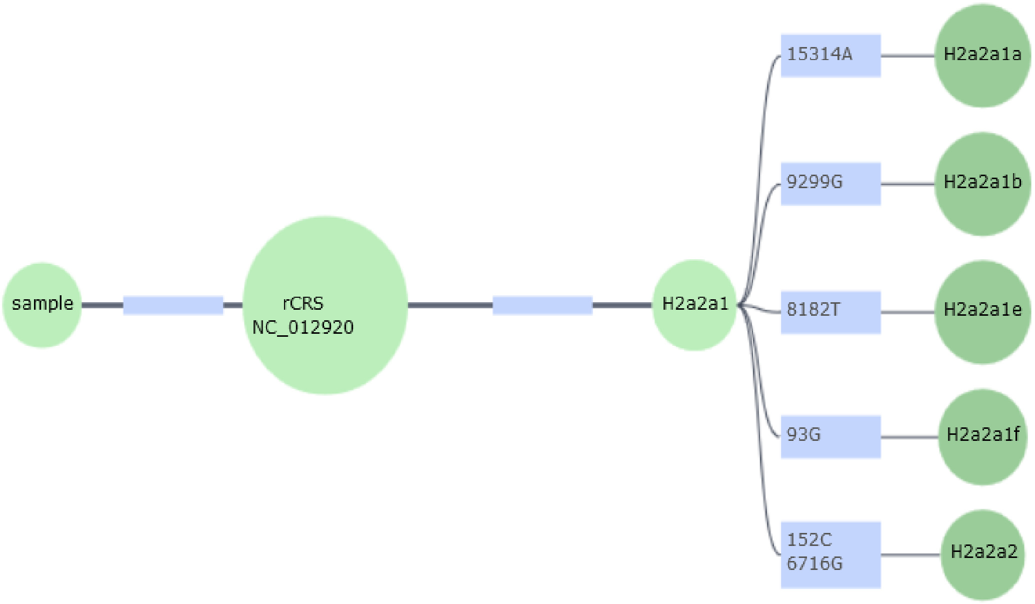

Fig. 1. Phylotree of haplogroups in mtDNA (control).

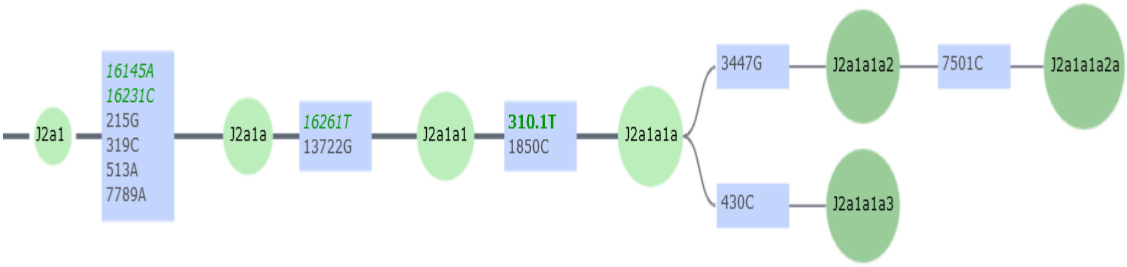

Fig. 2. Phylotree of haplogroups of mtDNA. samples G346 and G298

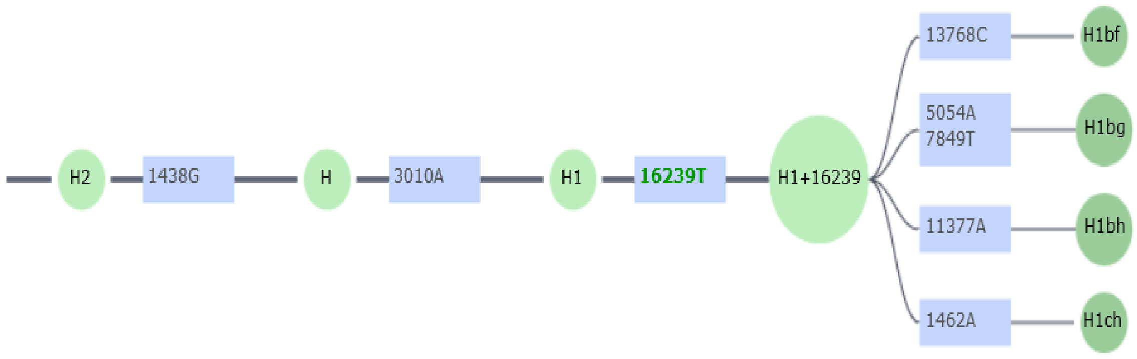

Fig. 3. Phylotree of haplogroups of mtDNA. Sample G361

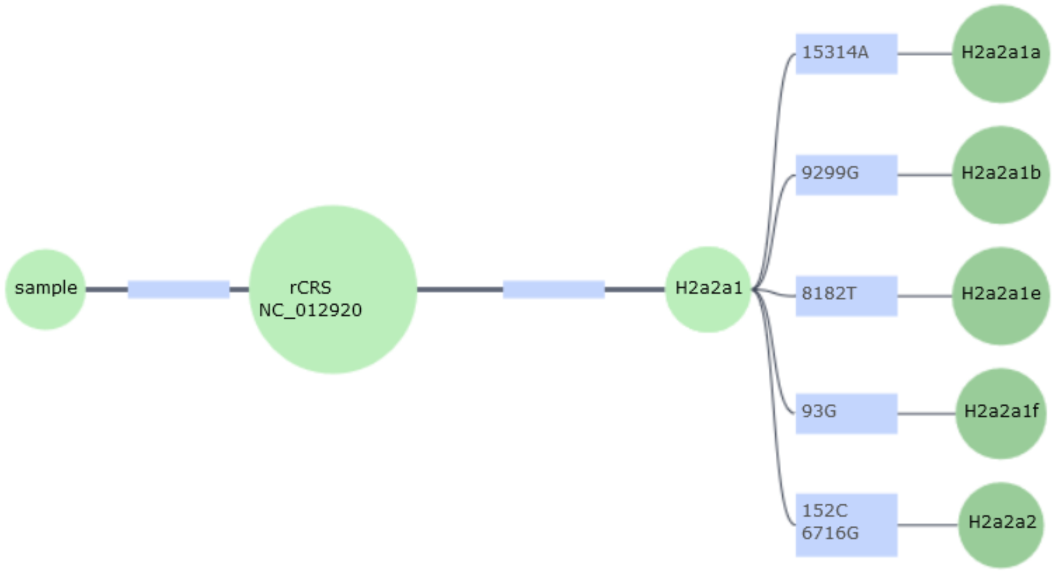

Fig 4. Phylotree of haplogroups of mtDNA. Sample G370

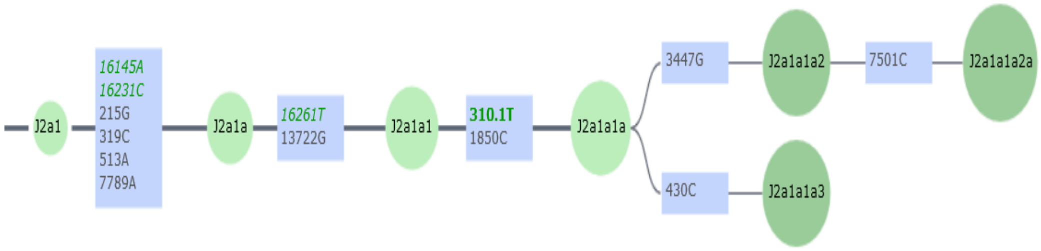

Fig 5. Phylotree of haplogroups of mtDNA (Research_worker)

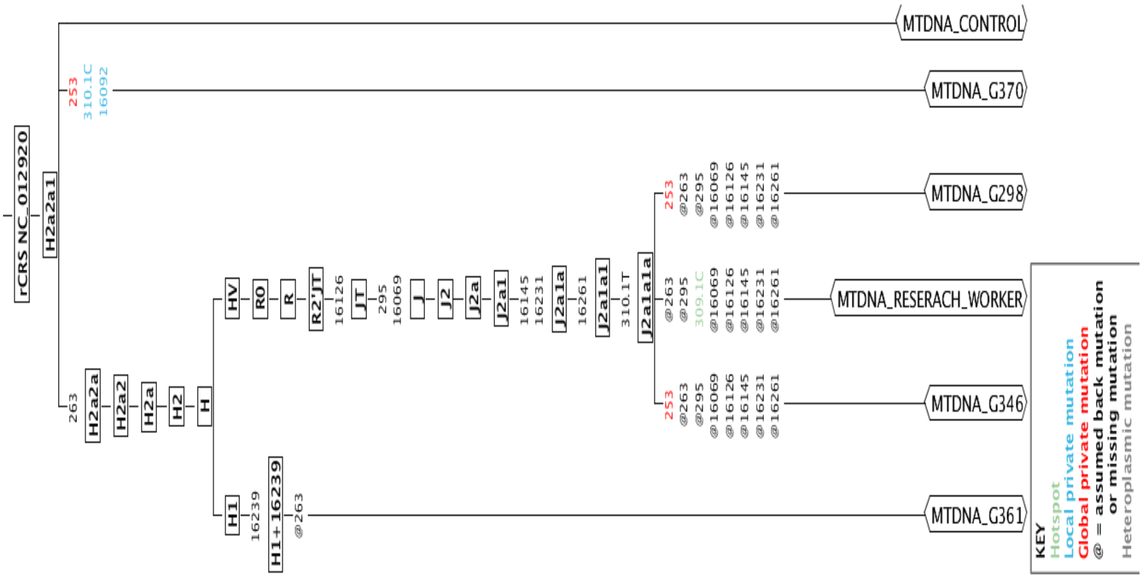

Fig 6 Phylogenetic tree of the haplogroups.
